# Laboratory confirmed miltefosine resistant cases of visceral leishmaniasis from India

**DOI:** 10.1186/s13071-017-1969-z

**Published:** 2017-01-31

**Authors:** Saumya Srivastava, Jyotsna Mishra, Anil Kumar Gupta, Amit Singh, Prem Shankar, Sarman Singh

**Affiliations:** Division of Clinical Microbiology and Molecular Medicine, Department of Laboratory Medicine, All India Institute of Medical Sciences, New Delhi, India

**Keywords:** Visceral leishmaniasis, Miltefosine, Drug resistance, Bihar, Jharkhand

## Abstract

**Background:**

Miltefosine unresponsive and relapse cases of visceral leishmaniasis (VL) are increasingly being reported. However, there has been no laboratory confirmed reports of miltefosine resistance in VL. Here, we report two laboratory confirmed cases of VL from India.

**Methods:**

Two patients with VL were referred to us with suspected VL. The first patient was a native of the VL endemic state of Bihar, but residing in Delhi, a VL non-endemic area. He was treated with broad-spectrum antibiotics and antipyretics but was unresponsive to treatment. The second patient was from Jharkhand state in eastern India (adjoining Bihar), another endemic state for VL. He was refractory to anti-leishmanial treatment, which included administration of miltefosine. Following investigation, both patients were serologically positive for VL, and blood buffy coat from both patients grew *Leishmania donovani*. The isolates derived from both cases were characterized for their drug susceptibility, genetically characterised, and SNPs typed for *LdMT* and *LdROS* gene expression. Both patients were successfully treated with amphotericin B.

**Results:**

The in vitro drug susceptibility assays carried out on both isolates showed good IC_50_ values to amphotericin B (0.1 ± 0.0004 μg/ml and 0.07 ± 0.0019 μg/ml). One isolate was refractory to Sb^III^ with an IC_50_ of > 200 μM while the second isolate was sensitive to Sb^III^ with an IC_50_ of 36.70 ± 3.2 μM. However, in both the isolates, IC_50_ against miltefosine was more than 10-fold higher (> 100 μM) than the standard strain DD8 (6.8 ± 0.1181 μM). Furthermore, genetic analyses demonstrated single nucleotide polymorphisms (SNPs) (_354_Tyr↔Phe and _1078_Phe↔Tyr) in the *LdMT* gene of the parasites.

**Conclusions:**

Here, we document two laboratory confirmed cases of miltefosine resistant VL from India. Our finding highlights the urgent need to establish control measures to prevent the spread of these strains. We also propose that *LdMT* gene mutation analysis could be used as a molecular marker of miltefosine resistance in *L. donovani*.

**Electronic supplementary material:**

The online version of this article (doi:10.1186/s13071-017-1969-z) contains supplementary material, which is available to authorized users.

## Background

Human visceral leishmaniasis (VL), commonly known as kala-azar, is a serious medical and public health issue in India, the Mediterranean region, and in parts of southern Europe, Africa, and South America where it is recognised as a neglected tropical disease [1, 2]. In India, the causative protozoan parasite is *Leishmania donovani* [3]. The parasite is transmitted *via* the bite of a female sand fly from the genera *Lutzomyia* and *Phlebotomus* [1, 3]. The clinical manifestation of leishmaniasis ranges from asymptomatic to fulminant that can progress to a highly fatal form, if untreated. VL is typically characterized by fever, weakness, progressive weight loss, pancytopenia, and massive hepato-splenomegaly [3].

The first line treatment of VL has been the pentavalent antimonials [sodium antimony gluconate (SAG)] [4]. However, in the last four decades, resistance to SAG has increased to alarming levels, particularly in some parts of India, forcing the national government to stop using of SAG [5]. Currently, the therapeutic options available for VL include amphotericin B (deoxycholate, liposomal or other formulations) and miltefosine [4]. Amphotericin B is highly effective in treating antimony-resistant cases [4] but its nephrotoxic side effects and the high cost of liposomal formulations restricts its wider use [6, 7]. Miltefosine, the first oral drug, was licensed for the treatment of VL in 2002 with reported cure rate of 98% [3, 5]. Currently, miltefosine is reported to be safe and well tolerated by adults and children [8] and plays a key role in India’s kala-azar elimination program, which aims to reduce the incidence of VL to less than 1 case per 10,000 in the next five years [9].

Recently, there have been an increasing number of cases of unresponsiveness to miltefosine and/or reduced efficacy in the treatment of VL in India and Nepal [9, 10]. Miltefosine is an alkyl-phospholipid, an analogue of phosphocholine. Its chemical similarity to the natural phospholipids of cellular membrane suggests that miltefosine probably inhibits transmembrane signals and the synthesis of the cellular membrane. The exact mode of its action and the developing resistance is not well known. The uptake of miltefosine in *Leishmania* spp. parasites involves the *Leishmania* membrane protein, *Leishmania donovani* miltefosine transporter (*LdMT*), a member of the P4 subfamily of P-type ATPases, and *LdRos*, a potential non-catalytic β subunit of *LdMT*. Both proteins are primarily localized in the *Leishmania* plasma membrane and are required for the rapid intracellular uptake of alkylphosphocholine drugs. *LdMT* and *LdRos* form a stable protein complex, which facilitates the translocation of phospholipids in the parasite from the exoplasmic sites to the cytoplasmic sites of the plasma membrane [11–14]. It is reported that even a single point mutation within the two alleles of the *LdMT* gene are responsible for the resistant phenotype through inactivation of the transporter protein [15, 16]. Consequently, mutational changes in *LdMT* (specifically, _856_Leu↔Pro, _420_Thr↔Asn, and _832_Leu↔Phe) have shown an increased rate of resistance in both in vitro and in vivo conditions [11, 12]. These mutations result in decreased uptake, increased efflux, faster metabolism [15, 17], and changes in the lipid composition of the parasite membranes [18]. Other mutations identified in the miltefosine resistant *L. donovani* include W210 (in *LdMT*) and M1 (in *LdRos*) [19] and single nucleotide polymorphisms (SNPs), _527_T↔A, which results in the substitution of _176_Val↔Asp in *LdMT* gene [20].

Although miltefosine resistant strains of *L. donovani* have not yet been cultured or isolated from the Indian subcontinent [10, 21], parasites with varying degree of miltefosine susceptibility have been reported [19]. In the absence of miltefosine resistant clinical isolates, the only option to study the mechanisms of miltefosine resistance has been to create in vitro resistant strains [14, 20, 22].

Recently, a case of VL was reported from Nepal with clinical relapse after successful treatment with miltefosine; however, the parasite could not be isolated from the patient [23]. Two cases of miltefosine unresponsive cutaneous leishmaniasis are also on record; one from Ecuador and another from Venezuela, but again without isolation of the parasite [24, 25]. Here we are reporting two cases of laboratory confirmed miltefosine resistant VL (*L. donovani*) from the VL endemic area of India with phenotypic and genotypic characterization of the isolates.

## Methods

### Patient history

**Fig. 1:**
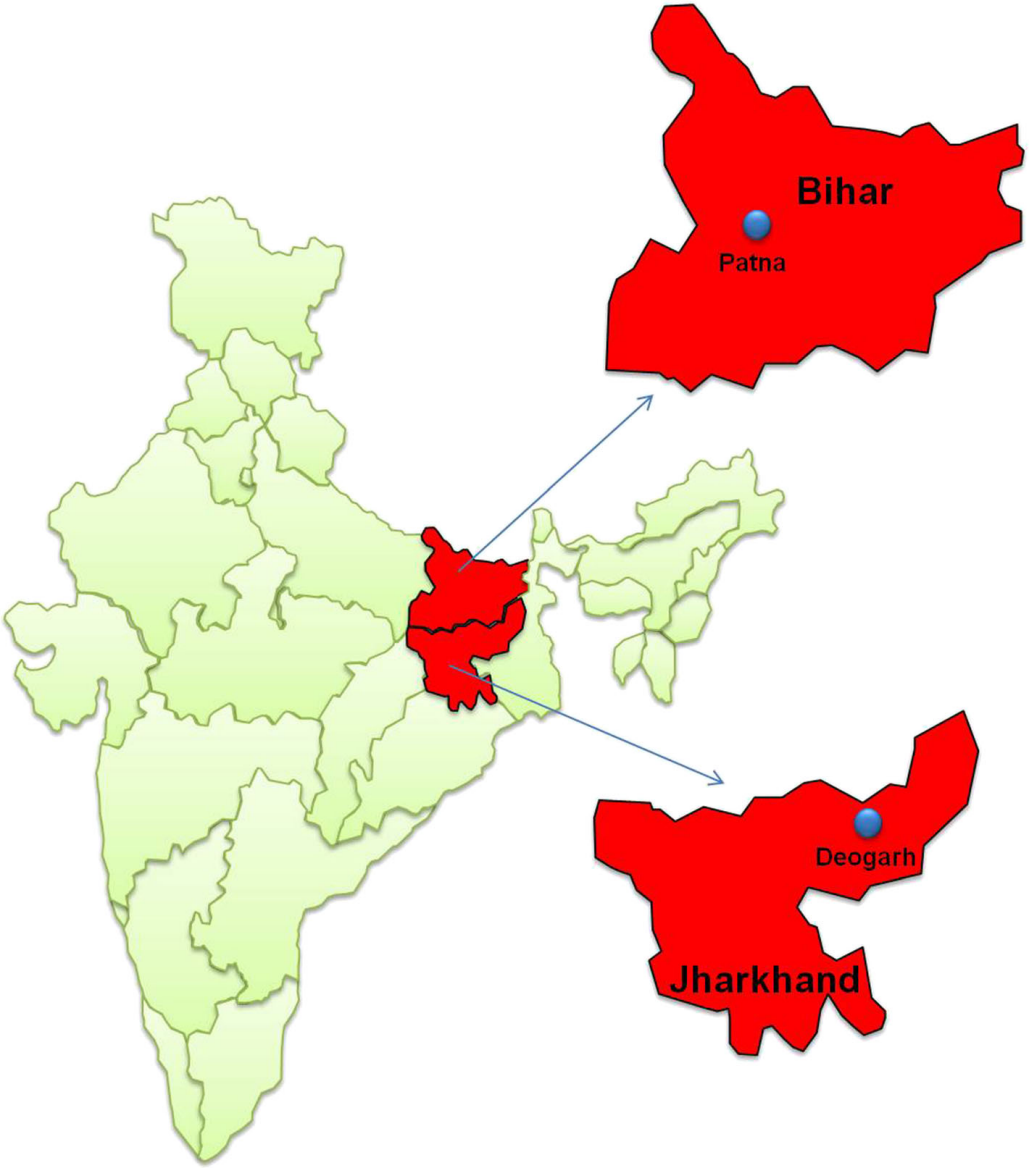
Map of India with highlighted VL endemic states of Bihar and Jharkhand (*red*). The map also shows the places from where two cases of miltefosine resistant VL are reported in this study

Two patients with suspected drug-resistant VL were referred to our laboratory for confirmatory diagnosis in the first 6 months of 2011. Both patients were from VL endemic areas. The first patient (LD843) was a 12-year-old boy native to the Patna district of Bihar (Fig. 1), but who had been residing in Delhi (a VL non-endemic area) for more than 5 years, although he frequently returned to his native village in Bihar. Referred in March 2011 from the Kalawati Saran Children Hospital, New Delhi, the patient had started having a high-grade fever for the past 20 days, which was not responding to antipyretics and broad-spectrum antibiotics (given locally). On clinical and laboratory examination, he was found to have moderate hepato-splenomegaly and was severely anaemic with haemoglobin levels of 5.2 g/dl [normal range (NR) 12–14 g/dl]. The patient had previously visited his native village in Bihar in November 2010.

The second patient (LD860), a 37-year-old man from the Deogarh District of Jharkhand (Fig. 1), was referred in April 2011. This individual presented at a local hospital in Jharkhand in November 2009, with complaints of loss of appetite and body weight. LD860 had a history of persistent fever for more than two weeks that was refractory to antipyretics and antibiotics. On clinical examination, the patient was anaemic, febrile, had moderate hepato-splenomegaly, with a palpable liver and spleen measuring 142 and 152 mm, respectively. He had a white blood cell count of 10,600 mm^3^ (NR 4,000–10,000 mm^3^), haemoglobin of 8.6 g/dl (NR 12–14 g/dl), with 50% neutrophils (NR 40–70%) and 44% lymphocytes (NR 20–35%). Following a positive serum aldehyde test for VL, in January 2010 SAG was administered at 20 mg/kg/day for a period of 21 days. Within 5 days his general condition improved and the patient became afebrile. However, after 6 months (August 2010) his clinical symptoms returned. At this point, he was severely anaemic with haemoglobin levels of 5.2 g/dl, leukocyte count of 8,800 mm^3^, with 63% neutrophils and 34% lymphocytes. Bone marrow aspirates showed an abundant number of Leishman-Donovan (LD) bodies. This time the patient was administered amphotericin B at 1.0 mg/kg/day for 15 days. However, treatment was interrupted due to poor tolerance. The clinical and laboratory signs of VL persisted and his repeat bone marrow remained positive for LD bodies. The patient’s liver enzymes were significantly increased with an SGPT value of 92 IU/l (NR 0–41 IU/l) and serum bilirubin of 2.6 mg/dl (NR 0.2–1.0 mg/dl). However, after cessation of amphotericin B treatment, his haemoglobin increased to normal limits i.e. 11.7 g/dl. In September 2010, miltefosine was administered to the patient at a dose of 50 mg/twice a day for 28 days. Although the patient became afebrile within a week and the laboratory findings were satisfactory, his liver enzymes and bilirubin levels remained high, for which he was given nutritional supplements and managed symptomatically. Again, in February 2011, the patient complained of fever, jaundice, and heaviness in the right subcostal region. Laboratory investigations revealed increased liver enzymes [SGOT] and reversed albumin-globulin ratio. Ultrasonography of the abdomen showed mild hepatomegaly and moderate splenomegaly. Finally, on 8th April 2011, the local physician referred the patient to the All India Institute of Medical Sciences, New Delhi, for further investigation and management.

Both patients underwent thorough laboratory investigations at the All India Institute of Medical Sciences, New Delhi. Sampling was conducted by the expert phlebotomist in the institute’s central collection facility and sent to us for routine investigations, including for leishmaniasis. The patient’s serum samples were tested by anti-rK39 dipstick test (kala-azar Detect™ Rapid Test, In Bios International, Inc., Seattle, WA, USA) and anti-rKE16 spot test (Signal^®^-KA, and Crystal^®^ KA, Arkray Healthcare Private Limited [formerly Span Diagnostics Limited], Surat, Gujarat, India The latter tests are based on a novel recombinant antigen [26] prepared from the Indian strain (KE16) of *L. donovani* (MHOM/IN/1998/KE16). Both are commercially available rapid tests and approved by the Government of India for confirmatory diagnosis of leishmaniasis. The Signal^®^-KA flow through spot test and Crystal^®^ KA (rKE16) lateral flow dipstick test and kala-azar Detect™ Rapid Test (rK39) [27] were strongly positive. The aetiological agent of disease was further confirmed by microscopic demonstration of LD bodies in smears prepared from blood buffy coat. A samples of blood buffy coat from both the patients were used for isolation of the parasite on Novy-MacNeal-Nicolle (NNN) medium.

### Parasite culture

To prepare NNN medium, 1.4 g of agar and 0.6 g of NaCl were suspended in 90 ml of distilled water, which was then autoclaved at 121 °C for 20 min. The solution was cooled to 50 °C before 10 ml of defibrinated rabbit blood was added. Two and a half ml of the mixture was then aliquoted into 7 ml Bijou tubes and allowed to cool and solidify at room temperature and finally stored at 4 °C. One aliquot was used for quality control. Just before inoculating the sample, 2 ml of M199 medium, supplemented with 20% v/v heat inactivated foetal bovine serum (FBS) and antibiotic solution containing 50 μg/ml gentamicin (Sigma-Aldrich, St. Louis, Missouri, USA) and 100 μg/ml of streptomycin sulphate salt (Sigma-Aldrich, St. Louis, MO, USA), was added. After that, buffy coat samples (washed two times with 1× PBS) were inoculated in NNN media and transferred to biochemical oxygen demand incubator (BOD) to incubate at 25 °C. Fungal contamination was checked after 4 days, and repeated every 4 days therefore to monitor the growth of *Leishmania* spp. until day 30th of inoculation. The culture was then passaged every seven days into fresh NNN medium. Parasites were then progressively adapted to medium M-199 supplemented with 10% FBS, gentamycin and streptomycin (complete medium) for mass culture. Each isolate was typed to the level of species using sequence data from the internal transcribed spacer (ITS) of DNA, following PCR (see below). The isolates were then characterized for their drug susceptibility. The parasites were tested for their in vitro susceptibility to standard anti-leishmanial drugs within ten passages from isolation. Both the isolates were subjected to nucleotide sequencing and SNPs analysis of *LdMT* and *LdRos* gene using Torrent variant caller software v5.0.1, as detailed below.

### Genetic characterization of species

Parasite DNA from both clinical samples were subject to PCR amplification of *Leishmania-*specific ITS regions. In brief, total genomic DNA was isolated from blood buffy coat using a DNeasy Blood & Tissue Kit (Qiagen, Hilden, Germany), as per manufacturer’s instructions. The complete ribosomal ITS region (~1.1 kb) was then amplified using *Leishmania*-specific primers (forward, 5'-CTG GAT CAT TTT CCG ATG-3'; and reverse, 5'-ACA CTC AGG TCT GTA AAC-3') [28]. The amplification was performed in the thermal cycler (Eppendorf, Hamburg, Germany) and initially heated to 95 °C for 2 min followed by 34 cycles of 95 °C for 20 s, 53 °C for 30 s and 72 °C for 1 min. The final extension was carried out at 72 °C for 1 min. The PCR amplicon was excised for purification using the QIAquick Gel Extraction Kit (Qiagen, Hilden, Germany) from a 1.8% agarose gel prepared in 1× TAE buffer, The purified PCR amplicon was then sequenced using the ABI Prism Big Dye Terminator v1.1 Cycle Sequencing Kit (Applied Biosystems, CA, USA).

### In vitro drug susceptibility test (MTT Assay)

The promastigote viability of the isolates (LD843 & LD860) compared to standard strain DD8, (routinely maintained and passaged in vivo in Syrian hamster to maintain virulence and infectivity) [29] was determined against 3 standard drugs using the MTT assay [30]. Cells (1 × 10^4^ cells/100 μl/well) were incubated in flat bottomed 96-well plate (Nunc, Roskilde, Denmark) using different concentrations of Sb^III^, an active component of SAG [4], amphotericin B and miltefosine for 48 h at 26 °C. The drugs were obtained from Sigma-Aldrich (Sb^III^), Lifecare Innovations Gurgaon, Haryana, India (Amphotericin B), and Zentaris Frankfurt, Germany (Miltefosine). In brief, stock solutions of all three drugs were dissolved in sterile distilled water. Each was further serially diluted up to seven-fold with freshly prepared complete medium and incubated as described above for 48 h at 26 °C in a BOD incubator. After 48 h of incubation, 25 μl of sterile MTT (5 mg/ml dissolved in 1× PBS) was added to each well and plates were incubated for 2 h at 37 °C. After incubation, 150 μl of pure dimethyl sulfoxide (DMSO) was added in each well to stop the reaction. The absorbance was measured in a microplate reader at 570 nm wavelengths. The inhibitory effect of the specific drug was expressed as 50% inhibitory concentration (IC_50_), i.e. the concentration of a drug which is required for 50% inhibition of cell viability compared to the control cells in culture medium without drugs. The IC_50_ was calculated from the graph showing different concentrations of the standard drugs plotted against percentage cell growth. Each test was performed in duplicate with three independent experiments.

### Anti-amastigote assay

The J-774A.1 mouse macrophage cell line was used to evaluate the activity of miltefosine against intracellular amastigotes transformed from clinical isolates. This was then compared against the WHO standard strain DD8, which is a pan-susceptible strain, isolated in 1968. Amastigote studies against SAG and amphotericin B were not conducted because the promastigote stage of clinical isolates was sensitive to these drugs. Cells were seeded in 16-well tissue culture chamber slides at a density of 4 × 10^4^ cells/ml in RPMI 1640 medium with 10% FBS. The slides were incubated at 37 °C with 5% CO_2_. After 24 h the medium was replaced with medium containing meta-cyclic promastigotes at a macrophage/parasite ratio of 1:10. This ratio allows promastigotes to be engulfed by macrophages and transformed into amastigotes with in 24 h. After 24 h of incubation at 37 °C with 5% CO_2,_one slide was fixed in 100% methanol and stained using Giemsa to determine the initial level of infection. Once a satisfactory level of infection was achieved, slides were exposed to miltefosine at doses of 100, 50, 25, 12.5, 6.25 and 3.12 μM (in duplicate) and incubated for 48 h at 37 °C and 5% CO_2_. Following incubation, slides were stained with Giemsa and microscopically examined. Parasite burden was calculated as [the percentage of infected macrophages × (mean number of amastigotes/macrophage)] and compared to the parasite burden in untreated infected control cells. Most of the macrophages were destroyed at higher concentrations of miltefosine (> 25 μM) and could not be evaluated. The results were calculated as the percent inhibition of the total parasite burden, and the IC_50_ was calculated from the graph showing different concentrations of miltefosine plotted against percentage inhibition. These tests were performed in duplicate with three independent repeats.

### Whole genome sequencing, assembly and SNPs analysis of LD843

For genome analysis, total genomic DNA (gDNA) was extracted from LD843 strain using the Qiagen Genomic DNA Extraction Kit (Qiagen, Hilden, Germany). The integrity and purity of gDNA was analysed on a 0.8% agarose gel prepared in 1× TAE. The whole-genome sequencing of LD843 strain was carried out using 2 × 400 bp paired-end reads using an Ion Torrent Personal Genome Machine (PGM, Thermofisher, MA, USA) and the sequencing chip 316v2 (Thermofisher, MA, USA). Low quality and adopter sequences were removed from the reads based on the phred quality score (Q-30) of individual bases. Reads were assembled into longer scaffolds using SPAdes assembly v3.1 [31]. Miscalled single bases and insertions/deletions (indels) in the assembled contigs were corrected with Iterative Correction of Reference Nucleotides (iCORN) software [32]. The sequence quality and read-depth coverage analysis were completed using Torrent coverage analysis v4.2 software [33]. The Integrative Genomics Viewer (IGV) [34] was used to visualize the individual reads for the analysis of read and mapping quality of contigs.

Non-mapping and replica reads were removed from the assembled reads based on lower mapping quality scores BQ (< 30). Sequencing analysis for LD860 is presently in process.

### SNPs analysis of the gene(s) involved in miltefosine resistance

SNPs analysis was carried out following alignment of the reference sequences for *LdMT* and *LdRos* gene sequences with the de novo aligned sequence of LD843 using Torrent variant caller software v5.0.1 [35] The regions which had a read depth of less than 100-fold coverage and a maximum of three polymorphisms in any given seven base regions were selected as candidate variable sites, while INDELs were identified with the Variant caller v5.1 software. The accepted read coverage of the variant bases were > 3 for forward and reverse strand and > 6 for SNP quality and minimum one distant read from contig gap. CIGAR scores displayed the mapping quality and SNPs for all reads at a specific site. For heterozygous SNPs, the variant BQ or CIGAR score was checked to make certain it was not considerably worse than the reference genotype BQ. To reduce sequencing error, a series of SNPs quality validation steps were adopted, including poor-quality SNPs detection. The position of individual SNP was visualized using the IGV software for the analysis of read depth, filtering quality, and haplotype score [34].

### Statistical analysis

The data are shown as the mean and standard deviation (± SD) of three independent experiments. The percent cell viability and IC_50_ values were calculated using Microsoft Excel 2007 (Microsoft Corp, WA, USA).

## Results

### Management of patients

Both patients received intravenous amphotericin B deoxycholate (1.0 mg/kg/day) for 15 days under strict medical supervision. LD843 was treated at the Kalawati Saran Children Hospital, New Delhi while LD860 was treated at the All India Institute of Medical Sciences, New Delhi. The treatment was successful with complete remission of the clinical symptoms within 2 weeks. Both patients were discharged from hospital. Their laboratory parameters also returned to normal within one month. The patients underwent routine medical checkups for 1 year following treatment. During this period neither of the patient reported relapse of symptoms.

### Characterization of the *Leishmania* spp.

**Fig. 2:**
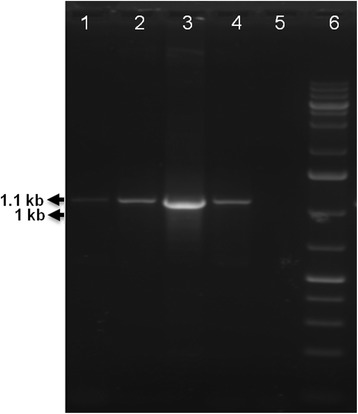
PCR analysis of buffy coat sample from serologically positive VL patients. Specific PCR product for ITS region of *Leishmania* spp. (~1.1 kb product; *arrow*). Lanes 1, 2: PCR amplified products of DNA extracted from the buffy coat of LD843 and LD860 patients respectively; Lane 3: positive control (DNA extracted from DD8 strain of *L. donovani* promastigotes); Lane 4: positive control (DNA extracted from the buffy coat of known positive patient); Lane 5: negative control (DNA from the buffy coat of healthy individual); Lane 6: 1 kb molecular weight DNA ladder

Parasite DNA from both clinical samples was subjected to PCR amplification (~1.1 kb) of the *Leishmania* specific ITS region (Fig. 2). Sequencing of these amplicons and subsequent comparison to publicly available sequence data indicated that sequences determined here showed 99% similarity with the *L. donovani* isolates (NICD/IN/50; accession number EU753224.2). Sequencing analysis, further confirmed that the cases were of *L. donovani* infection. The sequences obtained from the ITS region of DNA isolated from strain LD843 and LD860 have been submitted to GenBank under accession nos. JQ029056.1 and JQ780821, respectively.

### Drug susceptibility test

**Fig. 3:**
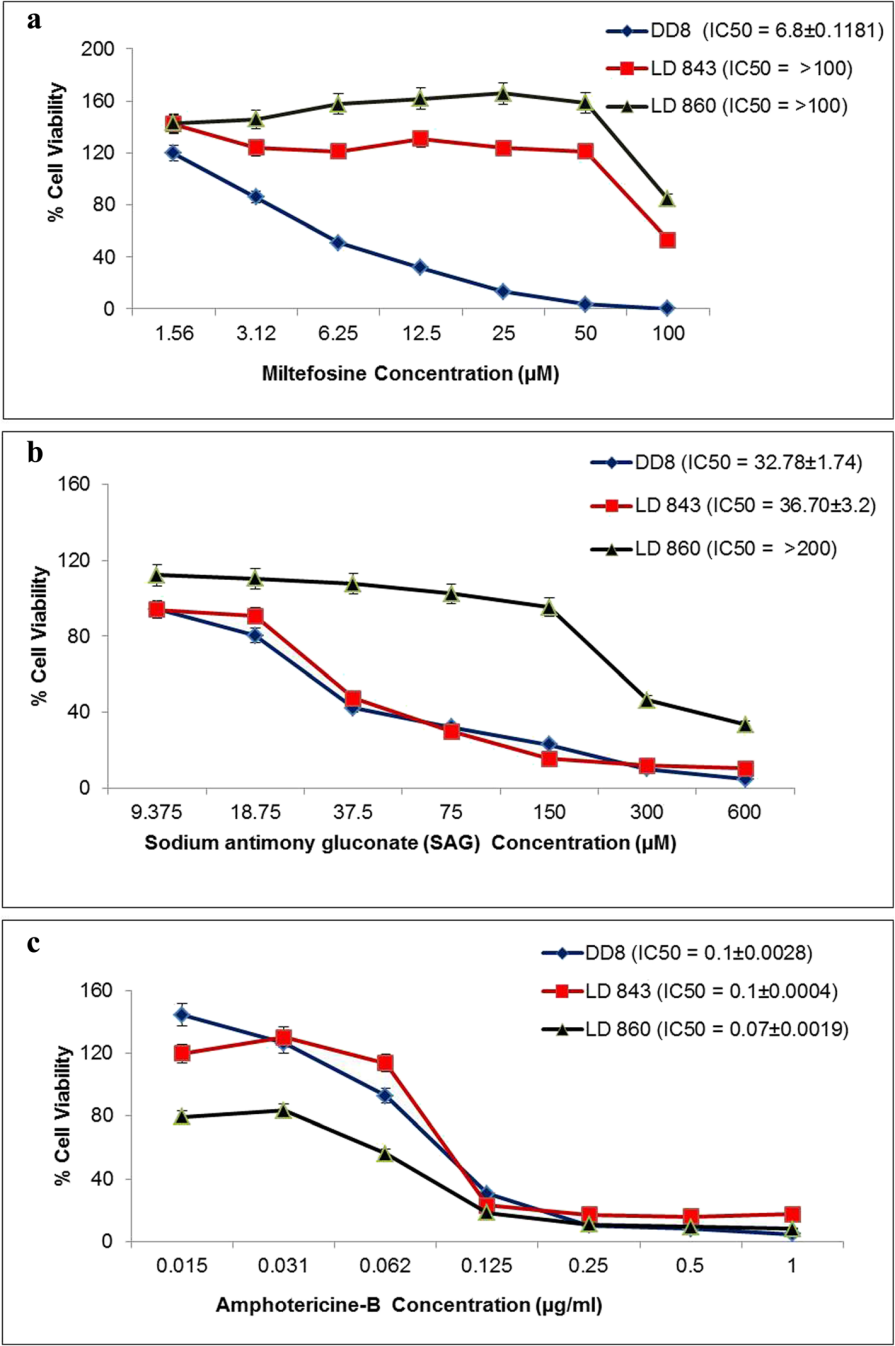
Representative plots of susceptibility profile (IC_50_) of parasite isolates from VL cases and standard strain DD8 to (**a**) Miltefosine, **b** SAG (Sb^III^) and (**c**) Amphotericin B. Each individual graph represents the mean from three separate assays

The IC_50_ for all three drugs (Sb^III^, amphotericin B and miltefosine) were determined by plotting drugs concentration *vs* percentage cell growth. The DD8 strain was found to be sensitive to all three drugs; IC_50_ values of 32.78 ± 1.74 μM, 0.1 ± 0.0028 μg/ml and 6.8 ± 0.1181 μM, respectively, were determined (Fig. 3). Both the clinical isolates, LD843 and LD860, showed good response to amphotericin B with IC_50_ values of 0.1 ± 0.0004 μg/ml and 0.07 ± 0.0019 μg/ml, respectively. However, both isolates showed > 10-fold increase in IC_50_ (> 100 μM) to miltefosine, compared to the DD8 strain (6.8 ± 0.1181 μM). In addition, LD860 was found to be resistant to Sb^III^ with an IC_50_ of > 200 μM, while LD843 was sensitive to Sb^III^ with an IC_50_ of 36.70 ± 3.2 μM (Fig. 3).

### Anti-amastigote assay findings

**Fig. 4:**
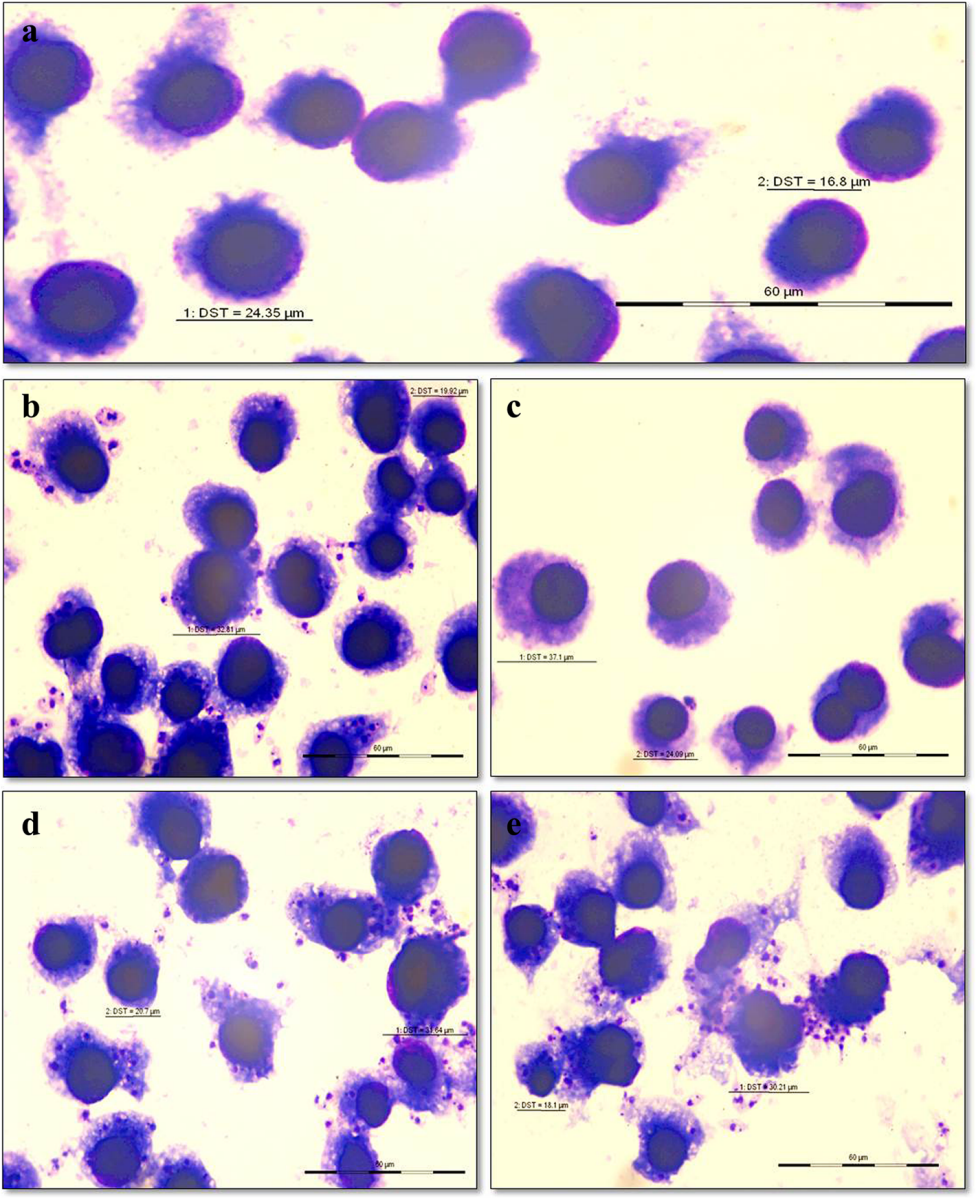
The *in vitro* miltefosine resistance demonstrated in clinical isolates using J774-A1 cell line. **a** Uninfected J774-A1 macrophages. **b** Macrophages cells infected with DD8 strain but untreated with miltefosine showing amastigotes within macrophages. **c** Miltefosine-treated DD8 infected macrophages showing clearance of amastigotes. **d** Macrophage cells infected with the clinical isolate but untreated with miltefosine and showing amastigotes within macrophages. **e** Macrophages infected with clinical isolate and treated with miltefosine showing no effect on the clearance of amastigotes. Giemsa stained macrophages cells were photographed at 1000× magnification using a light microscope

**Fig. 5:**
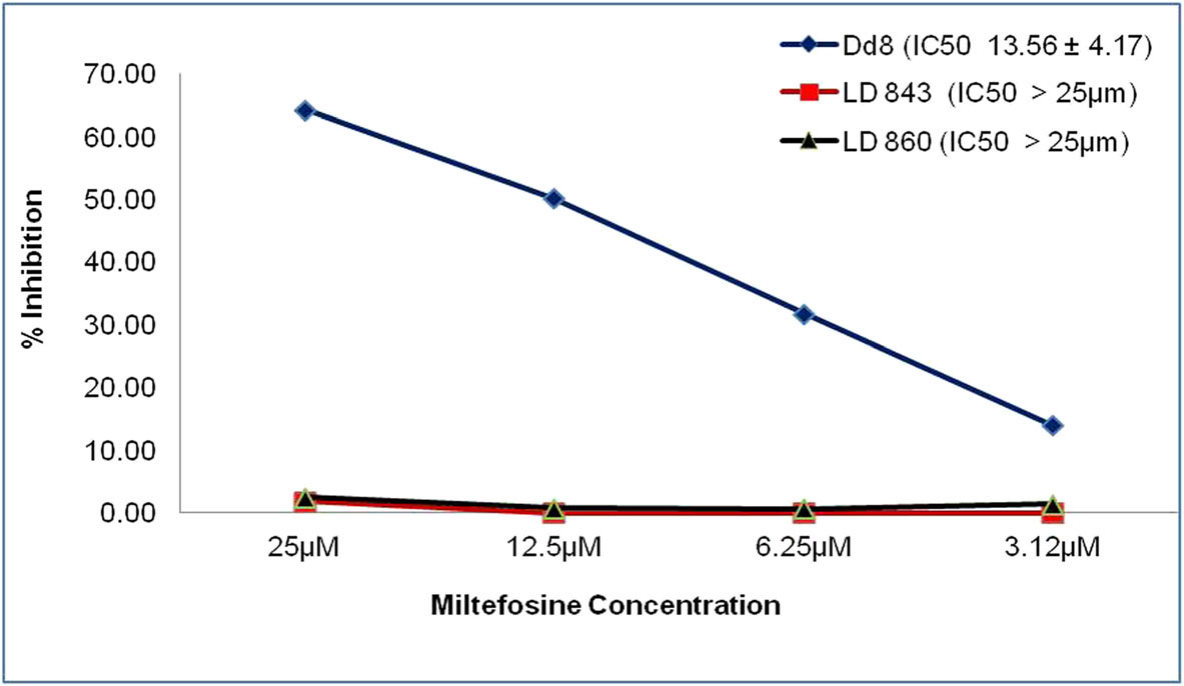
Representative plot of anti-amastigote assay of parasites isolated from kala-azar cases and standard strain DD8. The graph represents the % inhibition and mean IC_50_ of the results from three separate assays

All amastigote assays were carried out in the mouse macrophage cell line J-774A.1 (Fig. 4a). As the promastigote assays showed no resistance to Sb^III^ and amphotericin B, the amastigote assays were only performed using miltefosine. Like promastigotes, amastigotes also showed that miltefosine had no inhibitory effect on these clinical isolates. After 48 h, the percentage of parasites internalized by macrophages was higher in both isolates compared to those of the DD8 strain (Fig. 4b). After exposure of cells to miltefosine, the clearance of the internalized amastigotes was almost complete DD8 (Fig. 4c). Interestingly, the percentage of parasites internalized by macrophages was slightly higher in both the clinical isolates (Fig. 4d), but clearance of amastigotes in the miltefosine treated cells was negligible (Fig. 4e). Both the clinical isolates showed resistance to miltefosine and had an IC_50_ value of > 25 μM when compared to DD8, which had an IC_50_ value of 13.56 ± 4.17 μM (Fig. 5). All the experiments were repeated in triplicate. There were no significant differences between both the in vitro assays (*P* = 0.0485).

### Whole genome sequencing and assembly of LD843

The whole genome sequencing performed using an Ion PGM system produced a total genome length of 31,356,640 bp, or 31.35 Mb, at an average coverage of 23× depth (covering 99.76% of the genome), and a GC content of 56.68%. The *de novo* assembled genome of the LD843 strain of *Leishmania* consisted of 3,670 scaffolds, with the largest of these 137,235 bp, and an N50 of 21,796 and an N75 of 10,357 bp.

### SNPs analysis of gene involved in miltefosine resistance

**Fig. 6:**
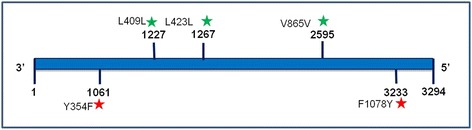
SNPs analysis of *LdMT* gene after genome sequencing of clinical isolates are indicated above the gene by star: *red* colour indicates non-synonymous mutations and *green* colour indicates synonymous mutations

A total of 31,356,640 nucleotide from the *de novo* assembled *Leishmania* genome (830.6× mean coverage depth of reference) were aligned to the reference sequences of *LdMT* (accession no. XM 003859320.1) and *LdRos* (accession no. FR799619.2) genes (Additional file 1). Twenty-one variants (indels) were detected in *LdMT,* while 13 were detected in *LdRos*. In-depth analysis of all variants conferred that five heterozygous SNPs were detected in *LdMT* whereas no significant SNPs were detected in *LdRos*. Three of the SNPs in *LdMT* were synonymous and two were non-synonymous. As synonymous SNPs do not affect the structural and functional activity of the protein, we only focused on the two non-synonymous SNPs (_1061_A↔T and _3233_T↔A),which resulted in the substitutions _354_Tyr↔Phe and _1078_Phe↔Tyr, respectively (Table 1, Fig. 6). The nucleotide substitutions are significant and perturb the protein’s structural stability. The nucleotide sequence of *LdMT* has been submitted to GenBank (KX827627).

**Table 1:** Gene mutations identified in miltefosine resistant strain LD843

S. No.	Chromosome no.	SNP type	Position in gene	Reference base	Reference cds	Base in LD843	LD843 cds	Amino acid change
1	13	Non-syn	1061	A	TAC	T	TTC	Y354F
2	13	Syn	1227	T	CTA	C	CTC	–
3	13	Syn	1267	T	TTG	C	CTG	–
4	13	Syn	2595	T	GTT	C	GTC	–
5	13	Non-syn	3233	T	TTC	A	TAC	F1078Y

*Abbreviations*: *S* serial; *SNP* single nucleotide polymorphism; *cds* codons; *Syn* synonymous; *Non-syn* non-synonymous

## Discussion

Visceral leishmaniasis is highly fatal if not treated [36]. In the absence of effective vaccines or vector control measures, the only option to control the disease is chemotherapy. For the last few decades, antimonials have been the predominant drugs of choice [4]. However, clinical resistance to these drugs emerged in the late 1980s and has become a principle obstacle in VL treatment and control [4, 36, 37]. Moreover, severe cardiac toxicity has further limited its use [38]. Alternative treatment options include amphotericin B and miltefosine. Both have similar efficacy, but miltefosine is an oral drug while amphotericin B requires intravenous infusion and hospitalization. The liposomal forms of amphotericin B are safer but prohibitively costly [6, 39].

Although miltefosine was the preferred treatment option for VL, with a cure rate as high as 98% [4], the long course of disease treatment, and the longer half-life (around 150 h), has rendered this drug highly vulnerable to the development of resistance [40]. This hypothesis is further supported by preliminary data of the post phase 4 trials of miltefosine showing the cure rate dropping to about 82–87% only [41]. Its vulnerability is further proved with the fact that miltefosine-resistant mutants of *Leishmania* can be generated very easily in vitro [42].

Although the exact method by which miltefosine resistance develops remains unknown, it is well documented that miltefosine uptake by *Leishmania* parasites involves multiple integral membrane proteins including *LdMT*, a member of the P4 subfamily of P-type ATPases, and *LdRos*, a potential noncatalytic β subunit of *LdMT* [11–14]. These trans-membrane proteins are required for the rapid intracellular uptake of alkylphosphocholine drugs such as miltefosine. It is conceivable that resistance may develop following the occurrence of non-synonymous point mutations in the genes coding for the respective proteins. Comparison of our genome sequence from strain LD843 with data publicly available for *LdMT* (3,294 bp) and *LdRos* (1,142 bp) revealed two novel SNPs in *LdMT*, _354_Tyr↔Phe and _1078_Phe↔Tyr. Tyrosine and phenylalanine play a key role in cell proliferation and differentiation via phosphorylation [43]. These non-synonymous SNPs in *LdMT* may cause conformational changes in this structural protein that may alter the plasma membrane and/or induced changes to miltefosine uptake by the parasite leading to the development of miltefosine resistance.

The present report highlights the existence of field strains of VL in India that are resistant to miltefosine, and provides laboratory confirmation of this finding. The high IC_50_ of the clinical isolates indicates in vivo selection of the miltefosine resistant clones. However, the paediatric case is of rather more concern as it indicates the possibility of transmission of the miltefosine resistant strain in the neighbouring area within a span of a few months only. The distance between Patna (Bihar) and Deogarh (Jharkhand) is approximately 300 km. Ultimately, this report sends a strong warning to VL control programs and WHO that miltefosine-resistant strains have emerged in India and that improved methods of treatment and control are required in order to prevent the spread of resistant strains to other areas within and outside India.

## Conclusions

Miltefosine was considered a highly effective oral drug for the treatment of VL in India, but these two cases of laboratory confirmed resistance in *L. donovani* against this drug sends an urgent signal for the need to search for new drugs or drug combinations. Although miltefosine induces an early clinical response in patients, increasing numbers of relapses indicate an urgent need for studying new drug development and use of combination therapies. As cell-mediated immunity against *Leishmania* parasites is compromised during the acute phase of VL, administration of miltefosine with agents that are known to have immunomodulatory, effects can be a good choice. We also propose that _354_Tyr↔Phe and _1078_Phe↔Tyr mutations in the *LdMT* gene can be used as molecular markers of miltefosine resistance in *L. donovani*.

## Additional file

Additional file 1: Sequence alignment and SNPs analysis of *LdMT* gene in LD843 strain (*LdMT*_M) with wild type sequence (*LdMT*_W). (PDF 268 kb)

## Abbreviations

DW: Distilled water
FBS: Foetal bovine serum
gDNA: Genomic DNA
IC_50_: 50% inhibitory concentration
ITS: Internal transcribed spacer
*LdMT*: *Leishmania donovani* miltefosine transporter
NR: Normal range
Phe: Phenylalanine
SAG: Sodium antimony gluconate
SNP: Single nucleotide polymorphism
Tyr: Tyrosine
VL: Visceral leishmaniasis
