# Supplementary material for: Laboratory confirmed miltefosine resistant cases of visceral leishmaniasis from India
Source: Parasit Vectors. 2017 Jan 31;10:49. doi: 10.1186/s13071-017-1969-z (PMC5282768; doi:10.1186/s13071-017-1969-z)
Supplement: Additional file 1: — Sequence alignment and SNPs analysis of LdMT gene in LD843 strain (LdMT_M) with wild type sequence (LdMT_W). (PDF 268 kb) [file 13071_2017_1969_MOESM1_ESM.pdf]

Additional File 1: Sequence alignment and SNP analysis of *LdMT* gene in LD843 strain (*LdMT*_M) with wild type sequence (*LdMT*_W)

```
LdMT_M      ATGCCCAACCAACCGCCGTGTTGGCGCAAGTGCCTTTCCACCAGAATCTTCCCAGACAAG 60
LdMT_W      ATGCCCAACCAACCGCCGTGTTGGCGCAAGTGCCTTTCCACCAGAATCTTCCCAGACAAG 60
            ************************************************************

LdMT_M      CTCTCCAAGTCCTTCTGCTGCTTTAGCGCAGAGGCGGACGTGGACGAGGATGATGTGGTG 120
LdMT_W      CTCTCCAAGTCCTTCTGCTGCTTTAGCGCAGAGGCGGACGTGGACGAGGATGATGAGGTG 120
            ******************************************************* ****

LdMT_M      ATCGTGTACCTTAACGACCCCGAGTTGAACGCGCAGTTTAATTATCCGTCGAACTTCATT 180
LdMT_W      ATCGTGTACCTTAACGACCCCGAGTTGAACGCGCAGTTTAATTATCCGTCGAACTTCATT 180
            ************************************************************

LdMT_M      CGTACCTCCAAGGACACACTCATCTCCTTCCTCCCACTCAGCCTCCTGTTGGAGTTCAAA 240
LdMT_W      CGTACCTCCAAGTACACACTCATCTCCTTCCTCCCACTCAGCCTCCTGTTGGAGTTCAAA 240
            ************ ***********************************************

LdMT_M      AAGGTGAGTAATTTGTATTTCCTCATGAACGTCATATTCAGCCTCATCCCAAGTGTGTCC 300
LdMT_W      AAGGTGAGTAATTTGTATTTCCTCATGAACGTCATATTCAGCCTCATCCCAGGTGTGTCC 300
            *************************************************** ********

LdMT_M      CCGCTAAGTCCGGCGACCTCGATTGCGCCGCTGTCCTTTGTGCTCATCGTGGCACTCATC 360
LdMT_W      CCGCTAAGTCCGGCGACCTCGATTGCGCCGCTGTCCTTTGTGCTCATCGTGGCACTCATC 360
            ************************************************************

LdMT_M      AAGGAGGGGGTGGAGGACATCAAGCGACATCAGGCCGACAACCGCGCCAACTCGATTTTA 420
LdMT_W      AAGGAGGGGGTGGAGGACATCAAGCGACATCAGGCCGACAACCGCGCCAACTCGATTTTA 420
            ************************************************************

LdMT_M      GTGCAGGTACTGCGAAACGGCAAGCTCGTCTCGGTGCACAGCAAGGACATCCACCCTGGT 480
LdMT_W      GTGCAGGTACTGCGAAACGGCAAGCTCGTCTCGGTGCACAGCAAGGACATCCACCCTGGT 480
            ************************************************************

LdMT_M      GACGTCATGCGTATCAAGAACGGCGAGGAGGTGCGCGCCGATGTCGTCATGCTCGCCTCG 540
LdMT_W      GACGTCATGCGTATCAAGAACGGCGAGGAGGTGCGCGCCGATGTCGTCATGCTCGCCTCG 540
            ************************************************************

LdMT_M      TCCGTCGAGGAAGGACAGGCATTTATAGACACATGTAACCTGGACGGCGAGACGAACCTG 600
LdMT_W      TCCGTCGAGGAAGGACAGGCATTTATAGACACATGTAACCTGGACGGCGAGACGAACCTG 600
            ************************************************************
```

```
LdMT_M      AAGTCGCGCAAGGCTCTGGAAGCCACCTGGGCGCTCTGCGAAGTCGAGGCAATCATGAAT 660
LdMT_W      AAGTCGCGCAAGGCTCTGGAAGCCACCTGGGCGCTCTGCGAAGTCGAGGCAATCATGAAT 660
            ************************************************************

LdMT_M      AGCACAGCCGTACTGCACACGAGCAAGCCAGACCCAGGGTTGCTGTCGTGGGCAGGGCTG 720
LdMT_W      AGCACAGCCGTACTGCACACGAGCAAGCCAGACCCAGGGTTGCTGTCGTGGGCAGGGCTG 720
            ************************************************************

LdMT_M      TTGGAAATCAATGGCGAGGAGCACGCTCTCTCGCTGAACCAGTTTCTGTATCGCGGCTGC 780
LdMT_W      TTGGAAATCAATGGCGAGGAGCACGCTCTCTCGCTGAACCAGTTCCTGTATCGCGGCTGC 780
            ******************************************** ***************

LdMT_M      GTGTGACGCAACACGGACTGGGTGTGGGGCATGGTTGCCTACGCAGGGGTCGACACGAAG 840
LdMT_W      GTGTTACGCAACACGGACTGGGTGTGGGGCATGGTTGCCTACGCAGGGGTCGACACGAAG 840
            **** *******************************************************

LdMT_M      CTGTTCCGAAACTTGAAGCCAAAACCGCCAAAGTCGTCGAACCTCGACCGCAAGCTGAAC 900
LdMT_W      CTGTTCCGAAACTTGAAGCCAAAACCGCCAAAGTCGTCGAACCTCGACCGCAAGCTGAAC 900
            ************************************************************

LdMT_M      TACTTTATCATAGCCGTCCTCATATTCCAGAACATCATGCTCTTCATCTTAGCCTCCATG 960
LdMT_W      TACTTTATCATAGCCATCCTCATATTCCAGAACATCATGCTCTTCATCTTAGCCTCCATG 960
            *************** ********************************************

LdMT_M      GCAGTGTGGTGGAACAGCAAGTACCGGGAAACGCCCTACCTCCGCTTCTTTATCAGCTTT 1020
LdMT_W      GCAGTGTGGTGGAACAGCAAGTACCGGGAAACGCCCTACCTCCGCTTCTTTATCAGCTTT 1020
            ************************************************************

LdMT_M      CGCAAGAACGCAACTCTGTGGGGATACCGCTACTTGAGTTTCTTCATTTTGCTGAGCTAC 1080
LdMT_W      CGCAAGAACGTAACTCTGTGGGGATACCGCTACTTGAGTTACTTCATTTTGCTGAGCTAC 1080
            ********** ***************************** *******************

LdMT_M      TGCGTGCCCATCTCGCTGTTCATCACGATTGAAGTGTGCAAAGTGGTCCAGGCGCAGTGG 1140
LdMT_W      TGCGTGCCCATCTCGCTGTTCATCACGATTGAAGTGTGCAAAGTGGTCCAGGCGCAGTGG 1140
            ************************************************************

LdMT_M      ATGCGGGTGGACTGCCTCATGATGGAGTACATGAGCAACCGCTGGCGGCACTGCCAGCCG 1200
LdMT_W      ATGCGGGTGGACTGCCTCATGATGGAGTACATGAGCAACCGCTGGCGGCACTGCCAGCCG 1200
            ************************************************************

LdMT_M      AACACGTCGAACCTCAACGAGCAGCTCGCAATGGTGCGCTTCATCTTCAGCGACAAAACT 1260
LdMT_W      AACACGTCGAACCTCAACGAGCAGCTAGCAATGGTGCGCTTCATCTTCAGCGACAAAACT 1260
            ************************** *********************************
```

```
LdMT_M      GGGACGCTGACAGAGAACGTCATGAAGTTTAAGCTAGGCGACGCTCTCGGTAATCCGATC 1320
LdMT_W      GGGACGTTGACAGAGAACGTCATGAAGTTCAAGCTAGGCGACGCTCTCGGTAATCCGATC 1320
            ****** ********************** ******************************

LdMT_M      GACGCCGACAATCTGGACGAGTGCATCGCGCAGCTGCGCAAGGAGGCCGAGTCGAAGGGG 1380
LdMT_W      GACGCCGACAATCTGGACGAGTGCATCGCGCAGCTGCGCAAGGAGGCCGAGTCGAAGGGG 1380
            ************************************************************

LdMT_M      CTAGGCCCGCTGCAAGAGTACTTTCTCGCGCTGGCCCTGTGCAACACGGTTCAGCCCTTC 1440
LdMT_W      CTAGGCCCGCTGCAAGAGTACTTTCTCGCGCTGGCCCTGTGCAACACGGTTCAGCCCTTC 1440
            ************************************************************

LdMT_M      AAGGACGACACGGATGACTTGGGTGTTGTCTACGAAGGCAGCTCCCCAGACGAGGTGGCG 1500
LdMT_W      AAGGACGACACGGATGACTTGGGTGTTGTCTACGAAGGCAGCTCCCCAGACGAGGTGGCG 1500
            ************************************************************

LdMT_M      CTGGTCGAGACCGCTGCTGCTGTTGGCTATCGCCTCATCAGCCGTACGACAAAGTCCATC 1560
LdMT_W      CTGGTCGAGACCGCTGCTGCTGTCGGCTATCGCCTCATCAGCCGTACGACAAAGTCCATC 1560
            *********************** ************************************

LdMT_M      ACGCTACTCCTGCACGATGGGACGCGCAAGGTATACAACATCCTCGCCACACTGGAGTTC 1620
LdMT_W      ACGCTACTCCTGCACGATGGGACGCGCAAGGTATACAACATCCTCGCCACACTGGAGTTC 1620
            ************************************************************

LdMT_M      ACGCCGGACCGCAAGATGATGAGCATCATCGTCGAGGACAGCGACACCAAAAAAATTACG 1680
LdMT_W      ACGCCGGACCGCAAGATGATGAGCATCATCGTCGAGGACAGCGACACCAAAAAAATTACG 1680
            ************************************************************

LdMT_M      CTGTACAATAAGGGGGCCGACAGTTTCATCAGGCCGCAGCTGAGCCGCGCCCCGGATGTG 1740
LdMT_W      CTGTACAATAAGGGGGCCGACAGTTTCATCAGGCCGCAGCTGAGCCGCGCCCCGGATGTG 1740
            ************************************************************

LdMT_M      CAGGGGCACATCGAAAATGTCGAGATCCCTCTGACGGAAATGTCCTCGTCGGGGCTCCGC 1800
LdMT_W      CAGGGGCACATCGAAAATGTCGAGATCCCTCTGACGGAAATGTCCTCGTCGGGGCTCCGC 1800
            ************************************************************

LdMT_M      ACGCTGCTTGTGTGCGCCAAGGAAATCACACGGCGCCAGTTCGACCCATGGTTCGAGAAG 1860
LdMT_W      ACGCTGCTTGTGTGCGCCAAGGATATCACACGGCGCCAGTTCGACCCATGGTTCGAGAAG 1860
            *********************** ************************************

LdMT_M      TTCGTCGAAGCCGGCAAGTCCCTGCACAACCGCAGCTCCAATATTGTTAAAGTCTGCTTA 1920
LdMT_W      TTCGTCGAAGCCGGCAAGTCCCTGCACAACCGCAGCTCCAATATTGATAAAGTCTGCTTA 1920
            ********************************************** *************
```

```
LdMT_M      GAGATGGAGCAAGATATGCGGCTCGTCGGTGCCACCGCTATCGAGGACAAGCTGCAAGAC 1980
LdMT_W      GAGATGGAGCAAGATATGCGGCTCGTCGGTGCCACCGCTATCGAGGACAAGCTGCAAGAC 1980
            ************************************************************

LdMT_M      GAGGTCCCTGAGACACTGTCCTTCTTCTTGAGCGCCGGTGTACTCATTTGGATGCTCACT 2040
LdMT_W      GAGGTCCCTGAGACACTGTCCTTCTTCTTGAGCGCCGGTGTAATCATTTGGATGCTCACT 2040
            ****************************************** *****************

LdMT_M      GGCGACAAGCGCGAGACCGCCGTGACGATCGCTGCAACGTCGACCCTGTGCGACCCGCGC 2100
LdMT_W      GGCGACAAGCGCGAGACCGCCGTGACGATCGCTGCAACGTCGACCCTGTGCGACCCGCGC 2100
            ************************************************************

LdMT_M      AACGACTTCATCGACCACATCGACATTGGTCATCTGAATTCATCGGATCCCAAGGCGATT 2160
LdMT_W      AACGACTTCATCGACCACATCGACATTGGTCATCTGAATTCATCGGATCCCAAGGCGATT 2160
            ************************************************************

LdMT_M      GAGCGCGTAGGGCGCGACCTCGAAGTGGTGGAGCAGCACATCGCGCTCAAGGGGACCCAC 2220
LdMT_W      GAGCGCGTAGGGCGCGACCTCGAAGTGGTGGAGCAGCACATCGCGCTCAAGGGGACCCAC 2220
            ************************************************************

LdMT_M      AAGGAGCGGCGCTGCACCTTGGTCATCGACGGCCCAGCGCTGAACATCGCAATGGAGCAC 2280
LdMT_W      AAGGAGCGGCGCTGCACCTTGGTCATCGACGGCCCAGCGCTGAACATCGCAATGGAGCAC 2280
            ************************************************************

LdMT_M      TACTTTGACCAGTTCCTGCGCCTCTCCCATCAGGTCAACTCCGCCGTCTGCTGTCGTCTC 2340
LdMT_W      TACTTTGACCAGTTCCTGCGCCTCTCCCATCAGGTCAACTCCGCCGTCTGCTGTCGTCTC 2340
            ************************************************************

LdMT_M      ACGCCGATCCAGAAGGCAACCGTCGTTCGCATGTTCCAGAAGTCAACCGGTAAGACAGCG 2400
LdMT_W      ACGCCGATCCAGAAGGCAACCGTCGTTCGCATGTTCCAGAAGTCAACCGGTAAGACAGCG 2400
            ************************************************************

LdMT_M      CTGGCCATCGGTGACGGCGCCAACGACGTGTCCATGATCCGGGAGGGGCGTGTGGGCGTG 2460
LdMT_W      CTGGCCATCGGTGACGGCGCCAACGACGTGTCCATGATCCGGGAGGGGCGTGTGGGCGTG 2460
            ************************************************************

LdMT_M      GGCATTATTGGGCTGGAAGGTGCACACGCCGCCCTCGCCGCCGACTACGCGATTCCGCGG 2520
LdMT_W      GGCATTATTGGGCTGGAAGGTGCACACGCCGCCCTCGCCGCCGACTACGCGATTCCGCGG 2520
            ************************************************************
```

```
LdMT_M      TTCAAGCACCTGCGCCGCCTATGCGCGGTGCATGGCCGCTACTCGCTCTTCCGCAACGCC 2580
LdMT_W      TTCAAGCACCTGCGCCGCCTATGCGCGGTGCATGGCCGCTACTCGCTCTTCCGCAACGCC 2580
            ************************************************************

LdMT_M      AGCTGCATTCTGGTCAGCTTCCACAAGAACAGTACTGTGTCGGTGGTGCAGTTCATCTTC 2640
LdMT_W      AGCTGCATTCTGGTTAGCTTCCACAAGAACATTACTGTGTCGGTGGTGCAGTTCATCTTC 2640
            ************** **************** ****************************

LdMT_M      GCCTTCTACGTCGGCTTCTCGGGGCTAACACTCTTTGATGGATGGATGCTGACCTTCTAC 2700
LdMT_W      GCCTTCTACGTCGGCTTCTCGGGGCTAACACTCTTTGATGGATGGATGCTGACCTTCTAC 2700
            ************************************************************

LdMT_M      AACGTCCTTCTAACAAGTATCCCACCCTTCTTCATGGGTATATTCGATAAGGACCTCCCC 2760
LdMT_W      AACGTCCTTCTAACAAGTATCCCACCCTTCTTCATGGGTATATTCGATAAGGACCTCCCC 2760
            ************************************************************

LdMT_M      GAAGATGCCCTGCTGGAGCGGCCGAAGCTGTACACCCCGTTGTCGCATGGCGAGTACTTT 2820
LdMT_W      GAAGATGCCCTGCTGGAGCGGCCGAAGCTGTACACACCGTTGTCGCATGGCGAGTACTTT 2820
            *********************************** ************************

LdMT_M      AACCTGGCGACGCTTCTGCGGTGGTTCGTCGAATCACTAACAACAGCGGTGATCCTCTTC 2880
LdMT_W      AACCTGGCGACGCTTCTGCGGTGGTTCGTCGAATCACTAACAACAGCGGTGATCCTCTTC 2880
            ************************************************************

LdMT_M      TATGCTGCTTACCCGACATTGATCCGTCAAGACGGTTCCCATCAGCGCTTCACCGGCGGC 2940
LdMT_W      TATGCTGCTTACCCGACATTGATCCGTCAAGACGGTTCCCATCAGCGCTACACCGGCGGC 2940
            ************************************************* **********

LdMT_M      GAGACCGGCACGCTCGTGTTCAGCGGCTTGATCCTCGTCATTCAAACTCGCTTCATCCTG 3000
LdMT_W      GAGACCGGCACGCTCGTGTTCAGCGGCTTGATCCTCGTCATTCAAACTCGCTTCATCCTG 3000
            ************************************************************

LdMT_M      CAGATCCGCTACTGGCAGTGGCTGCAGGTGTTTGGCATGGCGATGTCGATTTTTCTCTTT 3060
LdMT_W      CAGATCCGCTACTGGCAGTGGCTGCAGGTGTTTGGCATGGCGATGTCGATTTTTCTCTTT 3060
            ************************************************************

LdMT_M      CTGTTGTTGTTTCTCGTCTACTCCGCCATTCCCTCAGTCTTCAGTGACACGAATTTCGAC 3120
LdMT_W      CTGTTGTTGTTTCTCGTCTACTCCGCCATTCCCTCAGTCTTCAGTGACACGAATTTCTAC 3120
            ********************************************************* **

LdMT_M      TACCAAGCCTTCGATCTCATGTCGACCGCCAAGTACTGGTTTTTCCTGCTCCTCTACGTT 3180
LdMT_W      TACCAAGCCTTCGATCTCATGTCGACCGCCAAGTACTGGTTTTTCCTGCTCCTCTACGTT 3180
            ************************************************************
```

```
LdMT_M      GGCACCGAGGTGGTGGTCGTACTCGGCGTCATGACGTTCCAGAAGAACCTCTACCCTACC 3240
LdMT_W      GGCACCGAGGTGGTGGTCGTACTCGGCGTCATGACGTTCCAGAAGAACCTCTTCCCTACC 3240
            **************************************************** *******

LdMT_M      CTGCGCGACGTCGCGGAGCGACAGTACGCTGTTCAAAACGGTGGAAAGCTGTGA 3294
LdMT_W      CTGCGCGACGTCGCGGAGCGACAGTACGCTGTTCAAAACGGTGGAAAGCTGTGA 3294
            ******************************************************
```

Note: The alignment of nucleotide sequence (LD-843 strain) shows complete variants/SNPs obtained from Integrative Genomics Viewer (IGV). The validated /significant SNPs are highlighted as green colour (Synonyms SNPs), red colour (non-synonyms SNPs) and yellow colour (other non-significant SNPs).
